# NiO Pseudocapacitance and Optical Properties: Does The Shape Win?

**DOI:** 10.3390/ma13061417

**Published:** 2020-03-20

**Authors:** Marilena Carbone, Mauro Missori, Laura Micheli, Pietro Tagliatesta, Elvira Maria Bauer

**Affiliations:** 1 Department of Chemical Science and Technologies, University of Rome Tor Vergata, Via della Ricerca Scientifica 1, 00133 Rome, Italy; laura.micheli@uniroma2.it (L.M.); pietro.tagliatesta@uniroma2.it (P.T.); 2 Istituto dei Sistemi Complessi, Consiglio Nazionale delle Ricerche, Unit “Sapienza”, Piazzale Aldo Moro 5, 00185 Rome, Italy; mauro.missori@isc.cnr.it; 3 Istituto di Struttura della Materia, Consiglio Nazionale delle Ricerche (ISM-CNR), RM 1, Via Salaria km 29.3, 00015 Monterotondo, Italy; Elvira.Bauer@ism.cnr.it

**Keywords:** NiO, super-capacitance, optical properties, hydrothermal synthesis, porosity

## Abstract

In the present paper, we investigate the effects of alkali and operational temperature on NiO capacitive and optical properties. The NiO samples were prepared by a straightforward, surfactant-free hydrothermal synthesis, employing Ni(NO_3_)_2_ and either urea or moderately sterically hindered triethylamine (TEA). The syntheses were followed by calcinations at either 400 or 600 °C. NiO samples were characterized by XRD, scanning electron microscopy, and nitrogen adsorption isotherms. The optical properties were investigated by reflectance spectroscopy, and the pseudocapacitance was studied by cyclic voltammetry and galvanostatic charge charge-discharge measurements. We found that the synthesis with TEA yielded nanoflowers whereas the morphology of the synthesis with urea varied with the calcination temperature and resulted in nanoparticles or nanoslices at calcination temperatures of 400 and 600 °C, respectively. The NiO samples prepared at a lower temperature displayed a favorable combination of surface area and porosity that allowed for high performance with capacitances of 502 and 520 F g^−1^ at a current density of 1 A g^−1^ for nanoflowers and nanoparticles, respectively. The band gaps of all the samples were compatible with the estimated nanoparticle sizes. Finally, we used the synthesized NiO samples for the preparation of screen-printed electrodes (SPEs) modified by drop-casting and probed them against a [Fe(CN)_6_]^3−/4−^ probe.

## 1. Introduction

Energy harvesting and energy storage are preeminent issues, as fossil fuels are being depleted and alternatives become a necessity more than an aleatory option. When it comes to energy storage, Faradaic pseudocapacitors using oxidation-reduction reactions have the advantage over electrical double-layer capacitors (EDLC) of a higher capacitance, since the reactions occur both at the surface and near the surface of an active electrode [1]. In seeking materials for potential extensive applications, two aspects need to be balanced, high performances vs. a simple and cheap synthesis. With geometrical capacitance as an electronically driven factor, the structure and morphology of the capacitor material is an issue.

In this regard, NiO fulfills many of the desired characteristics, since it is cheap and widely used as the material of pseudocapacitors and can be synthesized via several routes.

NiO synthetic pathways include sol-gel, hydrothermal, solvothermal redox [2,3], and microwave-assisted reactions [4], among others. They can be obtained in several shapes, arrangements, and super-structures [5,6,7,8,9,10,11]. Although NiO properties can be tuned by targeted synthesis [12], the tuning procedure of the synthetic parameters is very fine, and only a narrow range of combinations yields one morphology or a specific range of pores sizes [13,14,15,16]. In the realm of simple synthesis, NiO can be obtained by the calcination of a precursor. The structural and morphological tuning can then be regulated by varying nickel salts or alkali used as the precipitating agents of the precursor and calcination temperature, time, as well as temperature ramp in the calcination phase. The influence of intercalating ions on the NiO pseudocapacitance was investigated by synthesizing Ni(OH)_2_ precursors via the hydrothermal procedure using urea and a surfactant cetyltrimethylammonium bromide (CTAB) as the templating agent and varying a Ni salt (Ni(NO_3_)_2_, NiCl_2_, or Ni(CH_3_CO_2_)_2_) [17]. Again, the effects of the synthesis on the NiO pseudocapacitance were investigated by varying the temperature of the hydrothermal procedure with Ni(NO_3_)_2_ and NH_4_HCO_3_ at a molar ratio of 1:2, followed by a calcination at 400 °C [18]. Different combinations of Ni salts and alkali were used by Kim et al. for preparation of NiO with different morphologies, and the corresponding pseudocapacitances were compared. In particular, the reaction of Ni(NO_3_)_2_ with hexamethylene tetramine yielded nanoflowers, whereas the synthesis using Ni(CH_3_CO_2_)_2_ and NH_4_OH or LiOH yielded nanoslices and nanoparticles, respectively [19].

Eventually, the properties are determined by a set, rather than a single morphological or structural parameter. Optical properties such as band gaps are smaller for nanoparticles as compared to those of bulk materials, and in general, the smaller the particle size, the smaller the gap [20], going down to a lower limit, where the trend is reversed due to confinement effects [21]. However, more parameters, such as the structural defects, impurities [22,23,24,25], and porosity [20], of nanoparticles play a role in determining band gaps, since they induce the delocalization of molecular orbitals in the conduction band edge, creating shallow/deep traps in the electronic energy and the boundaries of internal holes, which are also the sources of traps. Since morphology and size can be synthesis-controlled, a correlation has been proposed between the NiO solvothermal synthetic process and optical properties [26] as well as the calcination temperature [27]. 

Here, we investigated the effects of alkali and operational temperature on the capacitive and optical properties of NiO obtained by a straightforward, surfactant-free hydrothermal synthesis employing Ni(NO_3_)_2_ and either urea or moderately sterically hindered triethylamine (TEA). The syntheses were followed by the calcinations of precursors either at 400 or at 600 °C.

We found that the reaction with TEA yielded nanoflowers whereas the morphology of the synthesis with urea varied with the calcination temperature and resulted in nanoparticles and nanoslices at calcination temperatures of 400 and 600 °C, respectively. 

This approach has the advantage of being surfactant-free, hence leaving no trace of the templating agent throughout the steps of the synthesis; it is carried out in water, thus eliminating issues related to nonaqueous solvents and allowing for a direct correlation between preparation conditions and NiO morphology. Compared to previous preparations with analogous criteria [20], we opted for different precipitating agents, which favor either the formation of very small particles (urea) or the formation of flower-like structures (TEA), so as to compare the effects of the preparation on different types of morphology. In addition, we proceeded with a phased sonication, i.e., four sonication cycles of 15 min followed by 30 min breaks, to avoid the heating-up of the sonicated samples, hence aiding the “opening” of pores and channels.

The samples calcined at lower temperatures displayed a similar favorable combination of surface area and porosity, which corresponded to remarkably high capacitance performances. However, a distinction was made between samples treated at 600 °C, with nanoslices being better performing than large nanoflowers. The band gaps of all the samples were compatible with the estimated nanoparticle size. Finally, we used the synthesized NiO samples for the preparation of screen-printed electrodes (SPEs) modified by drop-casting. SPEs technology is widely used because it is handy, cheap, and miniaturization-oriented, with advantages of easy tunability towards specific electroactive targets via modification [28,29,30,31]. Nanosized NiO materials appear to be suited for the performances of SPEs modifications in terms of peak-to-peak separation and anodic and cathodic currents also being morphology-dependent [20,32,33]. Here, we examined the performances of the synthesized nanostructured NiO materials as agents to modify SPEs against a [Fe(CN)_6_]^3−/4−^ redox probe.

For the sake of clarity, it must be added that there is a debate going on, regarding the definition of the Faradaic pseudocapacitor. It has been argued that the cyclic voltammograms (CVs) of materials such as Ni(OH)_2_ showing distinct redox peaks should be classified solely as a faradaic battery-like material rather than a pseudocapacitor [34] whereas the definition of pseudocapacitors should be restricted to those materials such as RuO_2_, which undergo a redox reaction, without evident peaks in CV curves and linear charge-discharge curves [35]. On the other hand, it is considered that the distinctive difference between batteries and pseudocapacitive materials is that the charging and discharging of pseudocapacitive materials occurs on the order of seconds and minutes [36], regardless of the presence of peaks in CV curves.

NiO is among materials presenting redox peaks in CV curves and charging–discharging on the minute timescale, therefore lying in the “debated” zone. In this paper, we opted for defining the prepared NiO samples as pseudocapacitors, since they highlight a practical aspect of the material. We optimized the shapes and sizes of the NiO samples, which are typical parameters of capacitors, and finally the performances of these NiO samples were compared more straightforwardly to the literature data.

## 2. Experimental

### 2.1. Synthetic Procedure of NiO Nano- and Microstructures 

NiO samples were prepared via precursors synthesis followed by calcinations at either 400 or 600 °C. The different morphologies of the NiO samples were imparted through the employment of alkalis of different sterical hindrance, i.e., (CH_3_CH_2_)_3_N or (NH_2_)_2_CO, which reacted with Ni(NO_3_)_2_ to obtain precursors according to the reactions:

$$\mathrm{Ni}{\left({\mathrm{NO}}_{3}{)}_{2\;\left(\mathrm{aq}\right)}+2({\mathrm{CH}}_{3}{\mathrm{CH}}_{2}\right)}_{3}N_{\left(\mathrm{aq}\right)}+2H_{2}O\;\to \;\mathrm{Ni}{\left(\mathrm{OH}\right)}_{2\;\left(s\right)}+2{\left({\mathrm{CH}}_{3}{\mathrm{CH}}_{2}\right)}_{3}{\mathrm{NHNO}}_{3\;\left(\mathrm{aq}\right)}\;\mathrm{Ni}{\left({\mathrm{NO}}_{3}{)}_{2\;\left(\mathrm{aq}\right)}+({\mathrm{NH}}_{2}\right)}_{2}{\mathrm{CO}}_{\left(\mathrm{aq}\right)}+3H_{2}O\;\to \;\mathrm{Ni}{\left(\mathrm{OH}\right)}_{2\;\left(s\right)}+2{\mathrm{NH}}_{4}{\mathrm{NO}}_{3\;\left(\mathrm{aq}\right)}+{\mathrm{CO}}_{2}↑$$

Typically, 50 mL of a 1.2 mol L^−1^ aqueous solution of the chosen base were added dropwise to 50 mL of 0.6 mol L^−1^ Ni(NO_3_)_2_ aqueous solution at room temperature under vigorous stirring. The pH value was adjusted to 8.0 by using either a HNO_3_ or a NH_3_ solution. The suspension was then transferred into a 200 mL Teflon-lined stainless steel autoclave, carefully sealed and heated up at 180 °C for 24 h in a furnace. After gentle cooling, the obtained powder was repeatedly washed with deionized water and dried at 80 °C. The reaction yields were 99.6% for TEA and 99.2% for urea. Afterwards, the Ni(OH)_2_ precursors were placed in a tubular oven and heated up to the target temperature at a rate of 10 °C/min. Calcinations were carried out at either 400 or at 600 °C for 3 h in an air atmosphere. Four NiO samples were obtained, which were labeled A and U representing TEA or urea used in the synthesis, respectively, and 4 and 6 indicating the calcination temperatures of 400 or 600 °C, respectively. A scheme of the labeling is reported in Table 1.

Prior to characterization and electrochemical measurements, the synthesized samples were dispersed in ethanol and subjected to room-temperature-phased sonication, i.e., 4 cycles of 15 min sonication at 49 kHz followed by 30 min breaks, to avoid the heating-up of the sonicated samples. A quota of the dispersion was then directly deposited onto silicon wafers for SEM imaging, whereas the remaining dispersion was dried at 30 °C overnight.

### 2.2. Materials and Equipment

All chemicals were of reagent grade and used without any further purification. Ni(NO_3_)_2_, (CH_3_CH_2_)_3_N, and (NH_2_)_2_CO were purchased from Sigma-Aldrich. Ethanol and acetone were received from Merck. All solutions were prepared with deionized water. The XRD patterns of the synthesized samples were collected with an X’pert pro X-ray diffractometer by Philips (Almelo, The Netherlands), using Cu K-alpha radiation. The scanning electron microscopy images (SEMs) of the oxides were collected with a Zeiss Auriga field-emission scanning electron microscope (Jena, Germany) operating at 7 kV. Optical measurements in the ultraviolet (UV), visible (Vis), and near infrared (NIR) spectral regions were collected by a diffuse-reflectance setup from Avantes BV (Apeldoorn, The Netherlands). The latter comprised a combined deuterium-halogen radiation source (AvaLight-DH-S-BAL, Apeldoorn, The Netherlands) connected via an optic fiber to a 30 mm-diameter Spectralon®-coated integrating sphere (AvaSphere-30-REFL, Tampa, Florida). The sampling port of the sphere with a diameter of 6 mm was connected through another optical fiber to a spectrometer (AvaSpec-2048x14-USB2, Saint-Petersburg, Russia). This configuration allowed for applications in the 248–1050 nm range with a 2.4 nm spectral resolution. A laptop was used for spectrometer control and data recording, whereas BaSO_4_ was used as a reflectance reference. The specific surface area and porosity were measured on Micromeritics ASAP 2020 (Micromeritics Instrument Corporation, Norcross, Georgia, USA) using N_2_ adsorption–desorption isotherms. The NiO-based working electrode for pseudocapacitance measurements was prepared by mixing an 85 wt.% electroactive material with 10 wt.% acetylene black, and used 5 wt.% polyvinylidene difluoride (PVDF) as a binder. The mixture was then coated onto a Ni electrode and dried at 80 °C in an air atmosphere for 12 h. A Pt foil was used as a counter electrode, a KOH solution (2 M) was used as an electrolyte, and a saturated calomel electrode (SCE) was used as a reference. Galvanostatic charge-discharge and cyclic voltammetry were measured using a computer-controlled electrochemical interface (Solartron SI 1287, Durham, North Carolina, USA) and a potentiostat (Versa STAT 3, AMETEK, Durham, North Carolina, USA).

SPEs were fabricated by a precision screen printer DEK 245 (DEK, Weymouth, UK). Inks were purchased from Acheson Italia (Milan, Italy). The resulting SPE consisted of a graphite working electrode, modified by drop-casting of various NiO samples, a silver pseudo-reference electrode, and a graphite counter electrode and formed a complete electrochemical cell. Typically, the diameter of the working electrode is 0.3 cm, corresponding to an apparent geometric area of 0.07 cm^2^. CVs were recorded in a potential range between –1 and 1 V at a scan rate of 100 mV S^–1^, in 50 mM phosphate buffer + 0.1 M KCl, pH of 7.4.

## 3. Results and Discussion 

**Figure 1:**
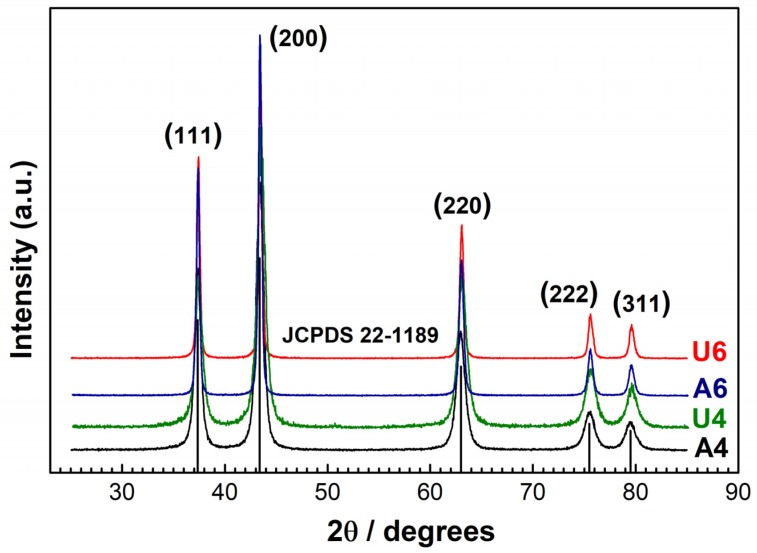
XRD diffraction patterns of NiO samples. The black solid line (**—**) represents sample A4, the green solid line (**—**) represents sample U4, the blue solid line (**—**) represents sample A6, and the red solid line (**—**) represents sample U6. The black vertical lines are the reflexes of NiO as from the reference data JCPDS 22-1189.

The structures of the NiO samples were analyzed by XRD. The diffraction patterns are reported in Figure 1 and correspond to a pure NiO phase [37]. The peaks at 2θ = 37.4°, 43.4°, 63.0°, 75.5°, and 79.6° were assigned to the (111), (200), (220), (222), and (311) reflections, respectively. The full width at half maximum (FWHM) varied, depending on the sample, and can be used to estimate the crystallites average size D, through the Scherrer equation described as: D = Kλ/βcos (θ), where K is a constant (ca. 0.9), λ is the X-ray wavelength used to collect XRD patterns (i.e., 1.5418 Å), θ is the Bragg angle, and β is the pure diffraction broadening of a peak at half height due to the crystallite dimensions. The estimated average crystallite diameters were about 10 ± 2 nm for samples A4 and U4 calcined at 400 °C and 25 ± 3 nm for samples A6 and U6 calcined at 600 °C.

**Figure 2:**
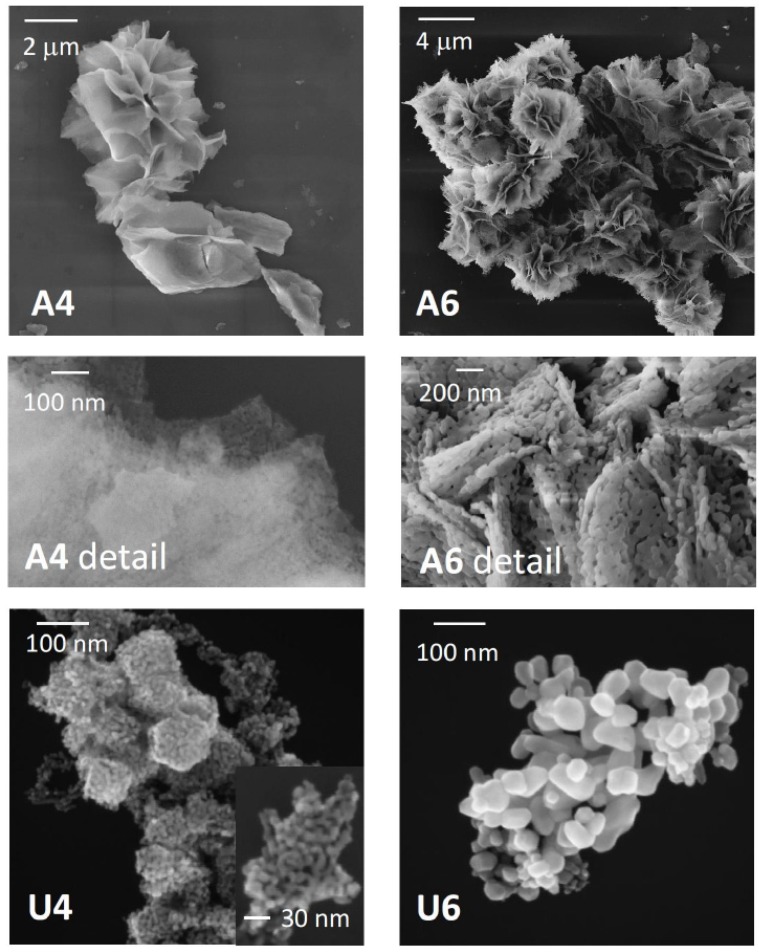
SEM images of different NiO samples. The samples are indicated by the labels in the figures. The central panels are the details of samples A4 and A6 at higher magnifications, where the texture can be better appreciated. The inset in the panel of sample U4 shows higher-magnification details, which evidences the nanoparticles size and aggregation.

Although the crystallite sizes of the samples calcined at the same temperature were comparable, the morphologies of the samples were very different, depending on the alkali used for the preparation. In Figure 2, the SEM images of the NiO samples are reported, showing flower-like microstructures for the specimens synthetized with TEA and nanoparticles or nanoparticles aggregates synthesized with urea.

The texture varied extensively among the samples, and differences of porosity can already be appreciated by the eye inspection of the high-magnification images. Indeed, in sample A4, the flower-like structure appeared to be generated by aggregates of grains, with an estimated size in a range of 10 nanometers, interconnected to form thin layers, which folded to give the characteristic floral appearance. Calcination at a higher temperature, such as in sample A6, had an effect on the microstructure, where more roses-like folds merged together to give a large envelop. On the nanometric level, since the grains are on average of larger dimensions, larger spaces were left between them (see the detail of sample A6, Figure 2). The type of aggregates was quite different when using urea in the synthesis. Small particles were obtained at 400 °C (sample U4) again in the 10 nanometer range, which did not show an overstructure, such as in sample A4, but rather aggregates in hexagonal shapes. 

When the sample was calcined at 600 °C (sample U6), nanoparticles with a large size dispersion and a nanoslice morphology were obtained, which did not aggregate according to a preferential pattern. A size statistic over more than 100 nanoparticles indicated an average size of 30 ± 20 nm.

**Figure 3:**
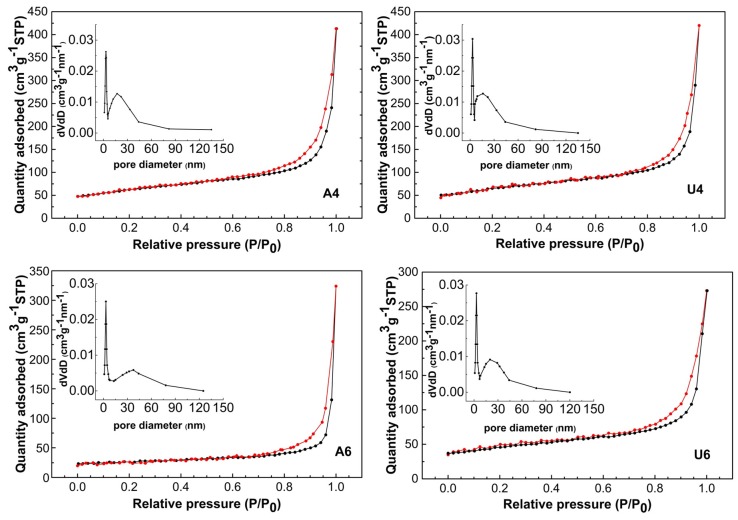
Nitrogen adsorption–desorption isotherms. The samples are indicated by the labels in the figures.

The surface area and porosity were determined by nitrogen adsorption–desorption isotherm measurements. The corresponding plots as well as Barret–Joyner–Halenda pore size distributions are reported in Figure 3. All isotherms were of type IV with narrow hysteresis loops at relatively low pressure, indicating fairly open mesoporous structure without significant obstruction to capillary evaporation vs. the condensation of nitrogen [38]. Samples A4 and U4 had the largest surface areas and comparable average pore sizes. Samples A6 and U6, in comparison, displayed a shrinkage of the surface area and an increase of the pore size at different extents. Surface area and porosity parameters are summarized in Table 2.

**Figure 4:**
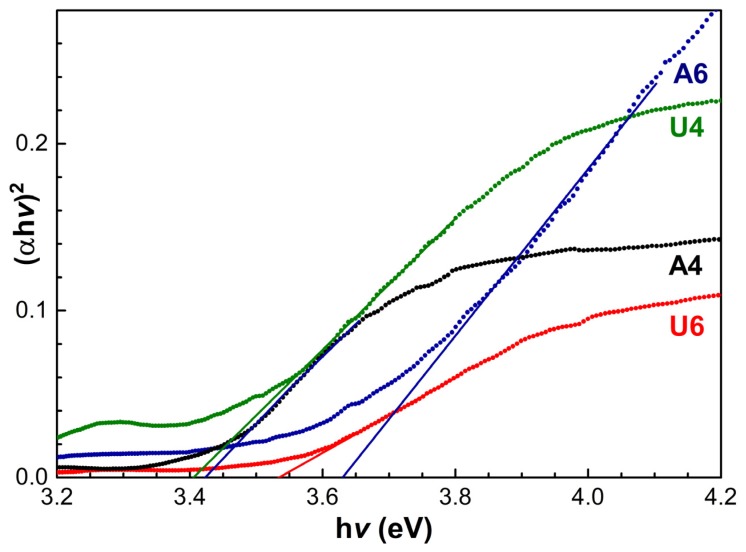
Tauc plots for the determination of the optical band gap E_g_ (dots) and associated extrapolations of the linear regions (solid lines). The black curve represents sample A4, the green curve represents sample U4, the blue curve represents sample A6 and the red curve represents sample U6.

The overall morphology had an effect on the optical properties of the samples. An evaluation was made by recording diffuse reflectance spectra R, which provided information if the particle size was comparable to or smaller than the incident wavelength of light and much smaller than the total thickness of the sample. In this case, reflectance spectra can be converted into absorption ones by using the Kubelka-Munk function [39,40]:

(1) $$\frac{K}{S}\equiv \frac{F{\left(1-R_{\infty }\right)}^{2}}{2R_{\infty }}\equiv F\left(R_{\infty }\right)$$

where *K* is the Kubelka-Munk absorption, *S* is the scattering coefficient, and $R_{\infty }$ is the reflectance of an infinitely thick layer of a sample. Therefore, preliminary tests were performed as the verification of the thickness of NiO powder in comparison to the well of the sample holder, which would allow us to consider $R_{\infty }$= *R* (infinitely thick sample). Furthermore, we can define the intrinsic absorbing coefficient of the particles *α* = *K/2,* in case of diffuse light distribution [41]. The steady preparation of the NiO powder sample for optical measurements also allowed us to consider the constant *S*, then the ratio *K/S* was approximately *α*. In order to estimate the band gap from the absorbance spectra, we then applied the Tauc relation [42]:

(2) $$\alpha hv=C1{\left(hv-E_{g}\right)}^{n}$$

where *n* = 2 is for direct gaps, *n* = 1/2 for indirect gaps such as that of NiO, C1 is constant, h is the Planck’s constant and *v* is the frequency of the electromagnetic radiation. The band gap E_g_ can be estimated by extrapolating the linear region of the ${\left(\alpha hv\right)}^{2}$ vs. h*v* plot (Figure 4). The values of band gaps and nanoparticle diameters obtained for the different NiO preparations are reported in Table 3. 

The nature of the trapping was recently investigated by D’Amario et al. [43], who generated electron–hole pairs in the band gap of NiO and followed the recombination dynamics. They associated the process with the generation of “Ni^3+^” and “Ni^4+^” ions, of which the lifetime may be affected by surrounding structures. In this regard, local structure and morphology are pivotal to the optical properties. Furthermore, nanoparticle boundaries can be both the sources of internal holes and the absorbers of traps. As a consequence, the shape, size, aggregation, and porosity of nanoparticles and microaggregates all play a role in the determination of band gaps. The band gaps we found were pretty much in line with these considerations, since they paralleled with the surface areas and porosities of the samples. Therefore, on the one side, we had the samples calcined at a lower temperature, i.e., samples A4 and U4, with gaps of 3.41 and 3.40 eV, respectively. Sample U6 with an intermediate surface area and an intermediate porosity displayed a higher value of a band gap, i.e., 3.53 eV, and sample A6, the least porous of the series, had the highest value, i.e., 3.63 eV. The band gap values also provided the estimated particle size, in terms of “equivalent” radius, i.e., spherical homogeneous particles, which would yield the corresponding band gaps, thus neglecting shape factors. This can be calculated by using the effective mass model with a Coulomb interaction term, where the bandgap E* (eV) can be approximated by:

(3) $$E^{*}≅E_{g}^{bulk}+\frac{\hbar^{2}\pi^{2}}{2er^{2}}\left(\frac{1}{m_{0}m_{h}}\right)-\frac{1.8e}{4\pi \varepsilon \varepsilon_{0}r}$$

where $E_{g}^{bulk}$ is the bulk band gap ($E_{g}^{bulk}\;$= 3.8 eV and $m_{h}\;$= 1.5 [44]), *r* is the particle radius, $m_{h}$ is the effective mass of holes in NiO,$\;m_{0}$ is the free electron mass, *ε* is the relative permittivity, *ε*_0_ is the permittivity of free space, ℏ is Planck’s constant divided by *2π*, and e is the charge of the electron [45]. The estimated values of the diameters (i.e., twice the calculated radii) are reported in Table 3, for an immediate comparison with the outcomes of the XRD estimates, along with the associated errors. They ranged between 11.2 ± 0.2 nm for sample U4 to 18.0 ± 1.4 nm for sample A6. The values for samples A4 and U4 were rather in line with the 10 ± 2 nm diameters, obtained by the Scherrer equation. The apparent discrepancies of samples A6 and U6 can be partly explained with the errors associated to the estimates with both techniques, as well as with shape factors, which play a role in opposite directions for these two techniques. With nanoslices, such as for sample U6, the shortest dimension was the limiting size of electron traps, whereas for XRD the largest dimension had a widening effect on the peak. 

**Figure 5:**
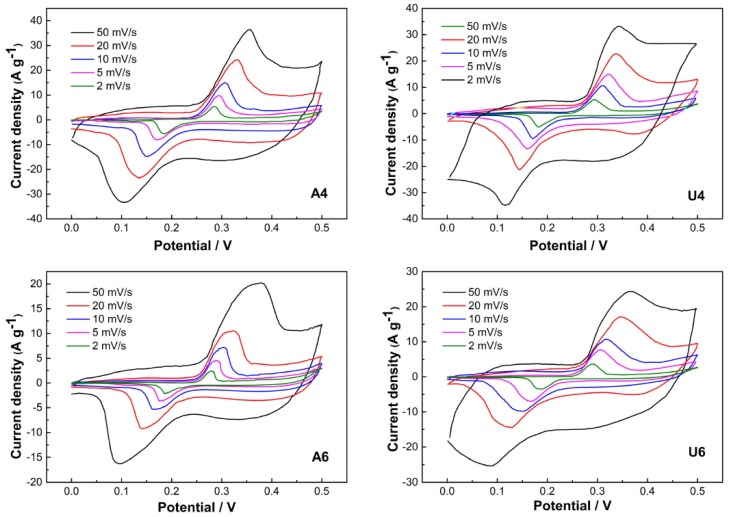
Cyclic voltammogram (CV) curves of the NiO samples in 2M KOH at various scan rates. The samples are indicated by the labels in the figures.

The morphology/structure-related pseudocapacitance was evaluated by cyclic voltammetry measurements of the four samples at different scan rates, as reported in Figure 5. The CV curves showed typical cathodic and anodic peaks arising during the redox reaction between NiO and NiOOH [17]:

$$\mathrm{NiO}+{\mathrm{zOH}}^{-}⇄\;\mathrm{zNiOOH}+\left(1-z\right)\mathrm{NiO}+{z\;e}^{-}$$

The noncomplete symmetry of anodic and cathodic sweeps indicated a nonthoroughly reversible process, as it can be expected for Faradaic redox reactions suffering polarization and ohmic resistance also playing a role due to electrolyte diffusion within a porous electrode [46].

The difference of the potentials between anodic and cathodic peaks can be viewed as a measure of the behavior of an electrode reaction. In this regard, samples U4 and A4 displayed the most reversible behavior (see Table 4), whereas sample A6 had the least reversible behavior.

It must be added that after cyclic voltammetry and in the presence of an electrolyte, NiOOH may be converted in Ni(OH)_2_ by the redox reaction:

$$\mathrm{Ni}{\left(\mathrm{OH}\right)}_{2}+{\mathrm{OH}}^{-}⇄\;\mathrm{NiOOH}+H_{2}O+{\;e}^{-}$$

An estimate of the specific capacitance can be deduced from CV curves by applying Equation (4):

(4) $$C_{s}=\frac{\int idV}{2vm\Delta V}$$

where C_s_ is the specific capacitance (F g^−1^), *i* is the cathodic or anodic instantaneous current (A), V is the instantaneous potential, *v* is the scan rate (V s^–1^), ΔV is the potential range, and m is the mass of the NiO material deposited on the unit surface of an electrode. The specific capacitance values were estimated to be 445, 440, 250, and 324 Fg^−1^ at a scan rate of 2 mV s^−1^ for samples U4, A4, U6, and A6, respectively, and they were reduced to 317, 305, 152, and 251 F g^−1^ at a scan rate of 50 mV s^−1^, due to the limited diffusion of OH^–^ inside the electrode material at faster OH^−^ production.

A more accurate specific capacitance value was obtained by galvanostatic discharge curves based on Equation (5):

(5) $$C_{m}=\frac{i*\Delta t}{m*\Delta V}$$

where *C_m_* is the specific capacitance, *i* is the charge-discharge current (A), Δ*t* is the discharge time of a cycle, m is the mass of NiO (g), and Δ*V* is the potential window (V).

**Figure 6:**
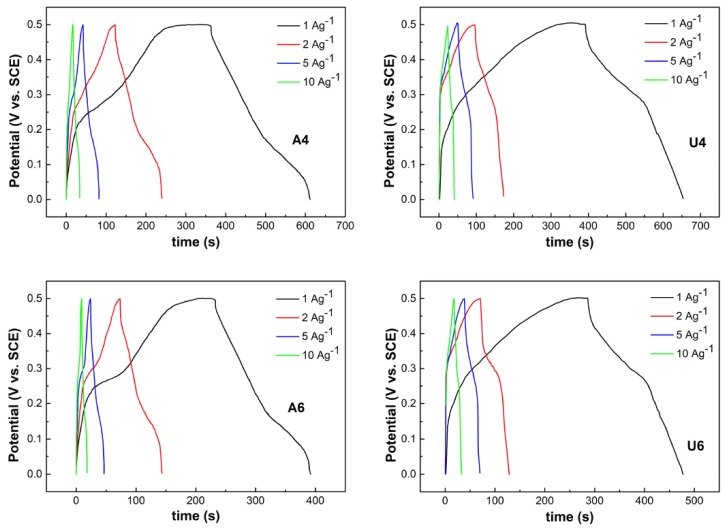
Galvanostatic charge-discharge curves of the NiO samples at various current densities. The samples are indicated by the labels in the figures.

The charge-discharge measurements were carried out in a 2M KOH solution between 0.0 and 0.5 V (vs. SCE) at various current densities ranging from 1 to 10 A^−1^ (Figure 6).

The specific capacitance values were in good agreement with the figures estimated by CV, which were 465, 475, 280, and 350 F g^−1^ at a current density of 2 A g^−1^ for samples A4, U4, A6, and U6, respectively, and reached 502, 520, 320, and 380 F g^−1^ at a current density of 1 A g^−1^. These values were higher than what were reported for NiO flakes [27], nanoflowers, nanoparticles, and nanoslices made according to a different preparation protocol [19]. We found that the lower-temperature preparations won over the higher-temperature ones, leading to better capacitors. There was no significant difference, however, between the low-temperature preparations of samples U4 and A4 in terms of capacitance, in spite of quite different morphologies. As for the higher-temperature preparations, sample A6 was flower-shaped like sample A4, but it had far worse performances; sample U6 was nanosliced and had an intermediate capacitance between those of samples A4 and A6. In the search for a common denominator through preparation methods and morphologies in correlation with the capacitance performance, the specific combination of characteristics such as the accessibility of reaction sites through pores and channels, along with the surface area, ultimately played a role in the charge storage. In a comparative study between flower-like, nanoparticles- and nanoslices-shaped NiO, the former displayed the largest capacitance, in spite of manifesting the smallest surface area among the three [19]. It was then hypothesized that the mesoporous structure of nanoflowers allowed for larger interparticles distances, thus facilitating three-dimensional networks and large electrolyte permeability. Furthermore, nanoflowers were more hydrous than the other shapes of NiO, allowing for an easier OH^−^ transport towards the inner sites; the pores of the structure also acted as an OH^−^ reservoir. The authors then concluded that the pore size is a more important parameter in determining the NiO capacitance. On the other hand, Zhao et al. investigated the capacitance properties of flower-like (flakes) morphologies of NiO as a function of calcination temperature and found lower performances for larger pores size and smaller surface areas, thus indicating that the surface area is more important [27]. Here, the finding is that the specific interplay of surface area and porosity/channels availability eventually determines capacitance properties, as they balance surface and subsurface processes. In this framework, one can evaluate the capacitance as the product of surface area and porous structure. The products were highest for samples A4 and U4, intermediate for sample U6 and lowest for sample A6, and the capacitance properties followed the same order. In terms of morphology, this can be seen as an equally performing situation, where crystallites of similar size (about 10 nm) were arranged in different superstructures but provided an analogous surface area/pore size balance (samples A4 and U4). A similar superstructure (sample A6) and a different one (sample U6) with a lower surface area/pore size product were also characterized by lower capacitance properties. A similar trend related to capacitance and surface size –pore size product can be envisaged in Kim et al. and Zhao et al. observations [19,27].

**Figure 7:**
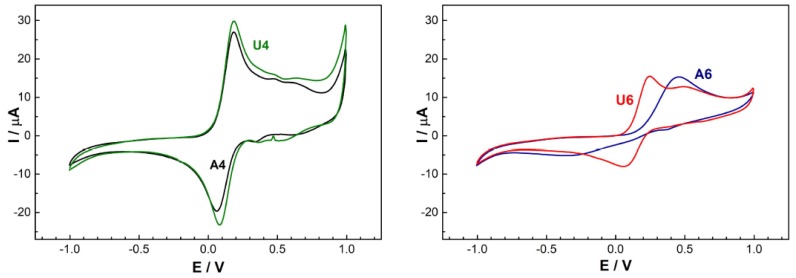
Cyclic voltammetries of the screen-printed electrodes (SPEs) modified with different NiO samples for the detection of a 20 mmol [Fe(CN)_6_]^3−/4−^ solution in 50 mM phosphate buffer (pH 7.4) with 0.1 M KCl. The samples are indicated by the labels in the figures.

The effect of the surface size-pore size product of NiO also reflected on the electrochemical performance in the detection of electroactive targets when deposited on SPEs. In Figure 7, the CV curves are reported for the detection of 20 mmol of [Fe(CN)_6_]^3−/4−^ in 50 mM phosphate buffer with 0.1 M KCl at pH of 7.4 and at a scan rate of 100 mV S^–1^. The anodic peak potential (E_pa_), cathodic peak potential (E_pc_), peak-to-peak separation ΔE_p_, anodic peak intensity (I_pa_), and cathodic peak intentsity (I_pc_) are summarized in Table 5. Here, again, the smallest peak-to-peak separations were achieved with SPEs modified with samples A4 and U4, and the largest peak-to-peak separation was realized by an SPE modified with sample A6.

Furthermore, samples A4 and U4 also had similar cathodic and anodic peaks intensities. Overall, the two samples displayed quite similar performances in spite of the different morphologies, thus indicating again that the specific surface area–pore size product plays a key role in determining electrochemical performances.

## 4. Conclusions

Four different samples of NiO were synthesized via a simple surfactant-free hydrothermal route, using Ni(NO_3_)_2_ and TEA or urea followed by calcination at 400 or 600 °C. Different morphologies were achieved, i.e., nanoflowers, nanoparticles, and nanoslices. The NiO samples prepared at a lower temperature displayed excellent performances with capacitance as high as 502 F g^−1^ (sample A4) and 520 F g^−1^ (sample U4) at a current density of 1 A g^−1^. Similar performances, in spite of the different morphologies of the samples, were explained in terms of specific surface area–pore size combinations, which accounted for the total surface and subsurface processes.

Reflectance measurements were performed, and they indicated smaller band gaps for the lower-temperature samples. The associated size of the crystallite was calculated, and it was in line with the X-ray estimate. Finally, we used the synthesized NiO samples for modifying SPEs and probed them against a [Fe(CN)_6_]^3−/4−^ redox probe and found a similar trend of performances to that of the charge storage.

## Figures and Tables

**Table 1:** Labels assigned to various NiO samples and preparation conditions.

Label	Alkali	Calcination Temperature
A4	(CH_3_CH_2_)_3_N	400 °C
A6	(CH_3_CH_2_)_3_N	600 °C
U4	(NH_2_)_2_CO	400 °C
U6	(NH_2_)_2_CO	600 °C

**Table 2:** Surface areas, pore sizes, and total pore volumes of the synthesized NiO samples.

Sample	Surface Area (m^2^ g^−1^)	Pore Size (nm)	Total Pore Volume (cm^3^ g^−1^)
A4	203.1	17.0	0.65
U4	210.3	16.7	0.66
A6	90.5	23.0	0.51
U6	160.1	19.6	0.43

**Table 3:** Band gaps estimated for the various NiO samples and the corresponding nanoparticles diameters with associated errors.

	Band Gap (eV)	Diameter (nm)
A4	3.41 ± 0.02	11.4 ± 0.2
U4	3.40 ± 0.02	11.2 ± 0.2
A6	3.63 ± 0.02	18.0 ± 1.4
U6	3.53 ± 0.02	14.0 ± 0.6

**Table 4:** Potentials for different NiO samples at a scan rate of 50 mV s^−1^. E_pa_ is the anodic peak potential, E_pc_ is the cathodic peak potential, and ΔE_p_ is the peak-to-peak separation.

Sample	E_pa_ (V)	E_pc_ (V)	ΔE_p_ (V)
A4	0.350	0.119	0.231
U4	0.342	0.117	0.225
A6	0.388	0.083	0.305
U6	0.361	0.081	0.280

**Table 5:** Summary of the electrochemical parameters of the SPEs modified with different preparations of NiO for the detection of 20 mmol [Fe(CN)_6_]^3−/4−^ solution in 50 mM phosphate buffer with 0.1 M KCl at pH of 7.4. The anodic peak potential (E_pa_), the cathodic peak potentials (E_pc_), the peak-to-peak separation (ΔE_p_), the anodic peak intentsity (I_pa_), and the cathodic peak intentsity (I_pc_) for each sample were obtained at a scan rate of 100 mV S^–1^.

Sample	E_pa_ (V)	E_pc_ (V)	ΔE_p_ (V)	I_pa_ (μA)	I_pc_ (μA)
A4	0.185	0.062	0.123	26.97	−19.65
U4	0.185	0.082	0.103	29.80	−23.23
A6	0.457	−0.361	0.818	15.32	−5.19
U6	0.246	0.056	0.190	15.49	−8.00
